# Role of Magnesium Deficiency in Promoting Atherosclerosis, Endothelial Dysfunction, and Arterial Stiffening as Risk Factors for Hypertension

**DOI:** 10.3390/ijms19061724

**Published:** 2018-06-11

**Authors:** Krasimir Kostov, Lyudmila Halacheva

**Affiliations:** 1 Department of Pathophysiology, Medical University-Pleven, 1 Kliment Ohridski Str., 5800 Pleven, Bulgaria; 2 Department of Physiology, Medical University-Pleven, 1 Kliment Ohridski Str., 5800 Pleven, Bulgaria; l_halacheva@abv.bg

**Keywords:** magnesium deficiency, arterial hypertension, vascular tone, arterial stiffness, vascular remodeling, insulin resistance, magnesium supplementation, dietary magnesium intake

## Abstract

Arterial hypertension is a disease with a complex pathogenesis. Despite considerable knowledge about this socially significant disease, the role of magnesium deficiency (MgD) as a risk factor is not fully understood. Magnesium is a natural calcium antagonist. It potentiates the production of local vasodilator mediators (prostacyclin and nitric oxide) and alters vascular responses to a variety of vasoactive substances (endothelin-1, angiotensin II, and catecholamines). MgD stimulates the production of aldosterone and potentiates vascular inflammatory response, while expression/activity of various antioxidant enzymes (glutathione peroxidase, superoxide dismutase, and catalase) and the levels of important antioxidants (vitamin C, vitamin E, and selenium) are decreased. Magnesium balances the effects of catecholamines in acute and chronic stress. MgD may be associated with the development of insulin resistance, hyperglycemia, and changes in lipid metabolism, which enhance atherosclerotic changes and arterial stiffness. Magnesium regulates collagen and elastin turnover in the vascular wall and matrix metalloproteinase activity. Magnesium helps to protect the elastic fibers from calcium deposition and maintains the elasticity of the vessels. Considering the numerous positive effects on a number of mechanisms related to arterial hypertension, consuming a healthy diet that provides the recommended amount of magnesium can be an appropriate strategy for helping control blood pressure.

## 1. Introduction

Magnesium (Mg^2+^) is the fourth most common mineral in the human body after calcium (Ca^2+^), potassium (K^+^), and sodium (Na^+^), and should be continuously replenished by food and water intake [1]. Mg^2+^ is the second richest intracellular cation after K^+^ and is a cofactor in more than 325 enzyme systems in cells [2]. Mg^2+^ is abundant in all green leafy vegetables, cereal, nuts, and legumes. Chocolate products, fruits, meat, and fish contain moderate amounts of Mg^2+^, and dairy products are low in Mg^2+^. Drinking water can be an important source of Mg^2+^ when it contains up to 30 mg/L of Mg^2+^ [3]. Chronic inadequate intake of Mg^2+^ over a long period of time can manifest as latent magnesium deficiency (MgD) [1]. Chronic MgD is associated with an increased risk of multiple preclinical and clinical manifestations including hypertension (HTN), atherosclerosis, cardiac arrhythmias, stroke, changes in lipid metabolism, insulin resistance, metabolic syndrome (MetS), type 2 diabetes (T2D), osteoporosis, depression, and other neuropsychiatric disorders [4] (Table 1). The assessment of Mg^2+^ status in the body is difficult because most is found in the cells or in the bones. Only 1% of the total Mg^2+^ in the body is present in extracellular fluids, and only 0.3% is found in the serum. The normal reference range for Mg^2+^ in the serum is 0.76–1.15 mmol/L [4]. In order to maintain these levels, the Recommended Dietary Allowance (RDA) for Mg^2+^ is 420 mg/day for men and 320 mg/day for women [5]. The hypomagnesaemia is defined as a condition where the serum concentration of Mg^2+^ in the body is <0.75 mmol/L [4]. When the Mg^2+^ intake is poor, the kidney can compensate by increasing reabsorption to >99% of the filtered amount, mainly in Henle’s loop and further in the distal tubules [1]. Signs and symptoms of hypomagnesemia usually occur when serum Mg^2+^ is decreased below 0.5 mmol/L. Many patients with hypomagnesemia remain asymptomatic [6,7].

Over the last eight decades, nutritional and serum Mg^2+^ levels have received more attention and have been the subject of comprehensive cardiovascular health studies. MgD is considered an important risk factor for various types of cardiovascular diseases (CVD) [8] (Table 1). Dietary studies in Europe and the United States of America (USA) reveal that Mg^2+^ intake is lower than recommended [4]. The often recommended daily intake for adults is 320–400 mg/day (or 6 mg/kg/bodyweight for both sexes) [1]. Epidemiological studies in Europe and North America show that people who consume Western-style diets have low Mg^2+^ content, <30–50% of the RDA for Mg^2+^. It is assumed that the Mg^2+^ intake in the USA has decreased over the past 100 years from about 500 mg/day to 175–225 mg/day. This is probably the result of the increasing use of fertilizers and processed foods [4]. Refining or processing of food may deplete Mg^2+^ content by nearly 85%. Especially boiling of Mg^2+^-rich foods can result in a significant loss of Mg^2+^ [3]. Furthermore, the incidence rate of MgD can vary considerably in different regions due to the large differences of Mg^2+^ content in drinking water, which can provide up to 30% of daily needs [1].

Therefore, it seems reasonable to assume that MgD is mainly related to the low intake of Mg^2+^ in food and drinking water, including the use of purified salt to cook, which may lead to a negative balance over time [1]. The processing and cooking of food may therefore explain the apparently high prevalence of low Mg^2+^ intake in many populations [3]. In the USA, the prevalence of insufficient Mg^2+^ intake in adults is about 64% among men and 67% among women. Among people over 71 years of age, the figure increases to 81% for men and 82% for women respectively [8]. In the USA the RDA, set at 320 and 420 mg/day for females and males respectively, is higher than the Reference Nutrient Intake of the United Kingdom (UK). Reported data for Mg^2+^ intake in UK compared to the USA RDA are significantly lower than recommended for all population groups. The UK general population’s mean intake is 270 mg and 221.4 mg for males and females respectively, representing only 64% and 69% of the US RDA [9]. Dietary intake of Mg^2+^ has also been shown to be insufficient in middle aged French adults, as 77% of women and 72% of men consume less than the recommended levels [10]. Furthermore, MgD may be potentiated by malabsorption or medication intake, such as diuretics (loop and thiazide), proton pump inhibitors, some antibiotics, and chemotherapeutic agents. With age, the absorption of Mg^2+^ from the intestine is reduced and its loss from the body in the urine increases in both sexes [1]. 

A number of literature reviews and editorials have focused on the importance of Mg^2+^ for the occurrence of CVD. These examinations have shown that the prevalence of cardiovascular disease events caused by an inadequate intake of Mg^2+^ and low serum concentrations of Mg^2+^, have been underestimated and cardiovascular health may be associated with the intake of Mg^2+^. A significant association was found between dietary Mg^2+^ intake and total cardiovascular disease risk. The greatest risk reduction was observed when dietary Mg^2+^ intake increased from 150 mg/day to 400 mg/day. Higher serum Mg^2+^ concentrations with 0.1 mmol/L were associated with a 9% lower risk for total cardiovascular events [8]. 

A significant part of the experimental studies link MgD and CVD, such as HTN, and atherosclerosis [4]. Given the increasing incidence of HTN, the identification of effective and safe preventative measures that offer even modest lowering of blood pressure (BP), could have a significant public health impact. In this regard, Mg^2+^ may have beneficial health effects for the primary prevention of HTN [11]. HTN is the largest contributor to the global burden of cardiovascular disease. The World Health Organization (WHO) estimates that the number of adults with high BP will increase from 1 billion to 1.5 billion worldwide by 2020 [12]. HTN is a major risk factor for heart disease and stroke [4]. According to WHO, 62% of all strokes and 49% of coronary heart disease events are attributable to high BP. Furthermore, experimental and epidemiological studies considered that HTN may serve as an effect modifier of the Mg^2+^ and CVD association [8].

## 2. Mechanisms Connecting MgD and Arterial Hypertension

**Figure 1:**
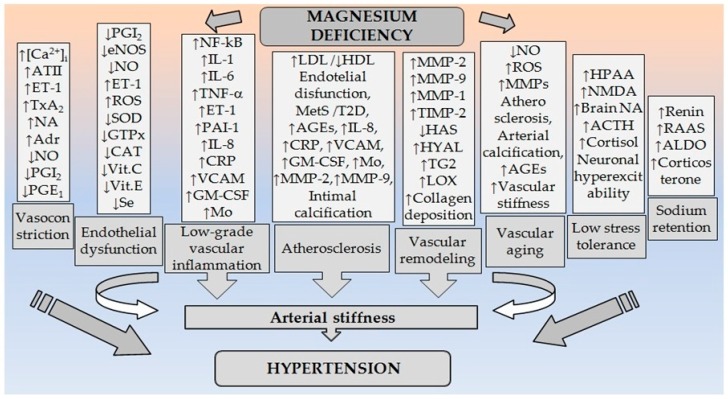
Pathogenetic relationships between MgD and arterial hypertension. (Abbreviations: ↑: increased; ↓: decreased; [Ca^2+^]_i_: intracellular calcium in VSMCs; ATII: angiotensin II; ET-1: endothelin-1; TxA_2_: thromboxane A_2_; NO: nitric oxide; PGE_1_: prostaglandin E_1_; PGI_2_: prostaglandin I_2_; ROS: reactive oxygen species; NF-kB: nuclear factor kappa B; TNF-α: tumor necrosis factor-α; IL-1: interleukin-1; IL-6: interleukin-1; IL-8: interleukin-8; PAI-1: plasminogen activator inhibitor-1; VCAM: vascular cell adhesion molecule-1; GM-CSF: granulocyte-macrophage colony-stimulating factor; Mo: monocytes; CRP: C-reactive protein; SOD: superoxide dismutase; GTPx: glutathione peroxidase; CAT: catalase; Vit.C: vitamin C; Vit.E: vitamin E; Se: selenium; LDL: low-density lipoproteins; HDL: high-density lipoproteins; MetS: metabolic syndrome; T2D: type 2 diabetes; AGEs: advanced glycation end products; MMPs: matrix metalloproteinases; MMP-1: matrix metalloproteinase-1; MMP-2: matrix metalloproteinase-2; MMP-9: matrix metalloproteinase-9; TIMP-2: tissue inhibitor of metalloprotease-2; HAS: hyaluronan synthases; HYAL: hyaluronidase; TG2: transglutaminase; LOX: lysyl oxidase; HPAA: hypothalamic pituitary adrenal axis; NMDA: N-methyl-d-aspartate receptor; NA: noradrenaline; Adr: adrenaline; ACTH: adrenocorticotropic hormone; RAAS: renin-angiotensin-aldosterone system; ALDO: aldosterone).

Mg^2+^ is involved in BP regulation. Every modification of the endogenous Mg^2+^ status leads to changes in vascular tonus, and as a consequence, changes in arterial BP [4]. Mg^2+^ plays an important role in BP regulation by modulating vascular tone and reactivity [13]. Small changes in both extracellular and intracellular Mg^2+^ concentrations have significant effects on vascular tone, contractility, reactivity, and growth [14,15]. MgD can increase BP by affecting multiple molecular and cellular mechanisms [16] (Figure 1).

### 2.1. Disturbances of Mg^2+^ Transport and Its Endocrine Control

Mg^2+^ transport occurs through two main pathways—transcellular and paracellular [5]. Transcellular transport includes influx and efflux transport systems. Mg^2+^ influx is controlled by a number of transporters such as mitochondrial RNA splicing 2 protein (Mrs2p), human solute carrier family 41, members 1 and 2 (SLC41A1 and SLC41A2), ancient conserved domain protein 2 (ACDP2), and Mg^2+^ transporter 1 (MagT1), as well as specialized cationic channels—transient receptor potential melastatin-6 and -7 (TRPM6 and TRPM7) cation channels [17]. TRPM6 channels are predominantly expressed in the kidneys and caecum, where they regulate Mg^2+^ reabsorption. TRPM7 channels are ubiquitously expressed and their absence is lethal [18]. Mg^2+^ efflux is accomplished by Na^+^-dependent and Na^+^-independent pathways [17]. Na^+^-dependent transport involves Na^+^/Mg^2+^ pump. The following participate in Na^+^-independent transport. The Mg^2+^/Ca^2+^ pump and Mn^2+^/Mg^2+^ antiporter Cl^−^/Mg^2+^ co-transporter. Paracellular Mg^2+^ transport is a passive process and takes place through dense intercellular contacts of the epithelial cells in the intestinal tract and the kidneys and depends on the specific structural proteins—claudins. Intestinal Mg^2+^ absorption is connected with the relatively low expression of ‘tightening’ claudins 1, 3, 4, 5, and 8. In the kidney paracellular Mg^2+^ transport depends mainly of claudin 16 (paracellin-1) and claudin 19 [5]. Disturbances of Mg^2+^ transport may predispose to the development of HTN and subsequent CVD [17].

The data reported up to now indicates the potential regulatory role of cation TRPM7 channels in maintenance of vascular integrity [19]. In vascular cells Mg^2+^ influx is mainly determined by the TRPM7 channels and disturbances in vascular smooth muscle cells (VSMCs) function in HTN can be partially linked to defective TRPM7 expression or activity [18]. TRPM7, implicated as a signaling kinase, is involved in a number of processes affecting VSMCs—growth, apoptosis, adhesion, cohesion, contraction, cytoskeletal organization, and migration. TRPM7 channels expressed in vasculature are regulated by vasoactive agents such as bradykinin, aldosterone (ALDO), endothelin-1 (ET-1), and angiotensin II (ATII) and different effects on the vascular wall such as pressure, tension, and osmotic changes. Thus these channels alter intracellular Mg^2+^ concentration by influencing the influx and efflux and may be associated with the onset and maintenance of HTN [17,18,20]. Experimental studies with Spontaneously Hypertensive (SHR) and Wistar-Kyoto (WKY) rats showed that TRPM6 and TRPM7 are differently regulated in their VSMCs. Inhibition of TRPM7, but not of TRPM6, may play a role in the altered Mg^2+^ homeostasis in VSMCs of SHR rats [21]. TRPM6 and TRPM7 are expressed in the endothelium, where they play an important role in intracellular Mg^2+^ homeostasis [22]. Because these channels have different effects on cell endothelial function it is suggested that they have potential importance, especially TRPM7, in the regulation of angiogenesis and vascular remodeling [23]. Mechanisms regulating vascular Mg^2+^ in health and disease remain unclear but TRPM7 could be important in HTN [20]. TRPM7 channels modulate endothelial behavior and any condition that leads to their increased expression, e.g., MgD or oxidative stress can damage endothelial function [19]. TRPM7 plays an important role in modulating VSMCs Mg^2+^ homeostasis, a major determinant of VSMCs function and vascular tone. Antunes et al. showed that in heterozygous TRPM7 kinase-deficient mice, ATII induces an exaggerated hypertensive response. These observations indicate that TRPM7 kinase differentially regulates vascular function in HTN. TRPM7 kinase is a modulator of BP regulation, which, when downregulated, promotes severe HTN and worsening of cardiovascular function. Moreover, ATII is a negative regulator of TRPM7. The study defines a novel TRPM7 kinase-sensitive mechanism involved in ATII-induced HTN. Based on these results, it can be concluded that aberrant TRPM7 expression/activity may contribute to impaired intracellular-free Mg^2+^ concentration and VSMCs contraction, proliferation, inflammation, and fibrosis, important determinants of vascular dysfunction and remodeling in HTN [20]. 

It has been proven that estrogens and epidermal growth factor (EGF) are magnesiotropic hormones [24,25,26,27]. Estrogens stimulate TRPM6 activity in short-term treatment and have long-term regulatory effects on TRPM6 transcription. The stimulation of a specific EGF receptor (EGFR) promotes TRPM6 trafficking to the plasma membrane. Long-term treatment with EGF regulates TRPM6 expression [28]. Considering the role of TRPM6 channels for changes of intracellular Mg^2+^ concentrations could be assumed that they are related to the modifications of the electrical and mechanical activity of cardiac myocytes or VSMCs and hence to CVD such as cardiac arrhythmias or HTN [29]. Thus, estrogen deficiency and the lack of their regulatory effects on Mg^2+^ exchange may be directly linked to increased loss of Mg^2+^ in postmenopausal women, which can lead to the development of HTN, cardiac arrhythmias, increased neuro-muscular excitability, and osteoporosis.

It is known that 1,25-dihydroxyvitamin D [1,25(OH)_2_D] can stimulate intestinal Mg^2+^ absorption [30]. In addition, conversion of vitamin D by hepatic 25-hydroxylation and renal 1α-hydroxylation into the active form and the vitamin D-binding protein are Mg^2+^-dependent. MgD leads to reduced 1,25(OH)_2_D and impaired parathyroid hormone (PTH) response. Absorption of Mg^2+^ and Ca^2+^ is inter-related. MgD impairs hypocalcemic-induced PTH release, which can be corrected after infusion of Mg^2+^. Mg^2+^ is also an important factor for the sensitivity of the target tissues to PTH. On the other hand, PTH has significant effects on Mg^2+^ homeostasis. For example, PTH release enhances Mg^2+^ reabsorption in the kidneys and absorption in the gut. PTH influences Mg^2+^ absorption, however, hypercalcemia antagonizes this effect [4]. The findings indicate a possible link between the vasculature and mineral metabolism. Epidemiological studies have shown that both PTH and Ca^2+^ are associated with high BP [31], which may be due to Mg^2+^ homeostasis disturbances.

### 2.2. Mg^2+^ as a Regulator of Vascular Tone and Reactivity

Mg^2+^ is one of the important physiological regulators of blood vessel tone. It improves vascular relaxation responses and attenuates agonist-induced vasoconstriction, thus mitigating the increased peripheral vascular resistance. The effects of Mg^2+^ as a regulator of vascular tone are most often the result of a competitive relationship with Ca^2+^. MgD increases the contractile response to various agonists and changes vascular responses to a number of vasoactive substances (Figure 1).

#### 2.2.1. Mg^2+^ as a Natural Calcium Antagonist

In the dissolved state, Mg^2+^ binds hydration water tighter than Ca^2+^. Thus, the hydrated Mg^2+^ is more difficult to dehydrate. The radius of hydrated Mg^2+^ is ~400 times larger than its dehydrated radius. This difference explains a lot of the biological properties of Mg^2+^, including its often antagonistic behavior to Ca^2+^, despite similar chemical reactivity and charge. For example, it is almost impossible for Mg^2+^ to pass through narrow channels in biological membranes, compared to Ca^2+^, because it cannot easily lose its hydration shell [32]. Because of its unique chemical properties, Mg^2+^ is linked to the modulation of intracellular Ca^2+^ homeostasis and decreased extracellular or intracellular Mg^2+^ can be combined with an increase in Ca^2+^ levels [16]. Ca^2+^ concentration in the cytosol is one of the principal factors determining the contractile properties of the VSMCs. Mg^2+^ counteracts Ca^2+^ and functions as physiological Ca^2+^ blocker, like synthetic Ca^2+^ antagonists [33,34,35]. Both extracellular and intracellular free Mg^2+^ can modulate VSMCs tone by voltage-dependent L-type Ca^2+^ channels. Extracellular Mg^2+^ inhibits Ca^2+^ current in VSMCs by two main mechanisms. First, extracellular Mg^2+^ effectively neutralizes the fixed negative charges on the external surface of the cell membrane. This stabilizes the excitable membranes and raises the excitation threshold which diminishes current via the voltage-gated Ca^2+^ channels. Second, some evidence suggests that extracellular Mg^2+^ can decrease Ca^2+^ current by directly binding to the Ca^2+^ channels. Binding of Mg^2+^ may either mechanically block the channel pore or may cause an allosteric modulation of the channel gating, resulting in its closure [36]. Elevated levels of extracellular Mg^2+^ inhibit Ca^2+^ influx, while decreased extracellular Mg^2+^ activates Ca^2+^ influx through Ca^2+^ channels [37]. Intracellular Mg^2+^ concentrations modulate VSMCs tone via its effects on ion channels and signal transduction pathways, especially those involving Ca^2+^. Changes in intracellular Mg^2+^ are known to influence these channels by affecting its amplitude, its activation/inactivation kinetics, and its modulation by factors such as phosphorylation, ultimately leading to decreased Ca^2+^ entry. Intracellular Mg^2+^ activates sarcoplasmic/endoplasmic reticular Ca^2+^ ATPase pump that sequesters intracellular Ca^2+^ into the sarcoplasmic reticulum. Elevated intracellular Mg^2+^ stimulates inositol-1,4,5-trisphosphate (IP3) breakdown, inhibits IP3-induced Ca^2+^ release from the sarcoplasmic reticulum, and competes with intracellular Ca^2+^ for cytoplasmic and reticular binding sites [36]. Low intracellular Mg^2+^ stimulates IP3 mediated mobilization of Ca^2+^ from the sarcoplasmic reticulum and reduces Ca^2+^ ATPase activity, decreasing Ca^2+^ efflux and reuptake by the sarcoplasmic reticulum. This leads to accumulation of cytosolic Ca^2+^, increased intracellular Ca^2+^ concentration, which is a crucial factor for vasoconstriction [37]. Mg^2+^ can also block Ca^2+^ release from the sarcoplasmic reticulum via the ryanodine receptor [17]. An important property of Mg^2+^ is to compete with Ca^2+^ for binding sites on regulatory molecule troponin C. Thus, Mg^2+^ regulates the activity of contractile proteins and their dynamics [16]. Lastly, intracellular Mg^2+^ regulates activity of the G-protein-coupled receptors as AT1 (ATII), ETA (ET-1), V1a (vasopressin), and α_1_-adrenoceptors (norepinephrine and epinephrine) on VSMCs and intracellular Ca^2+^ signal transduction pathways as translocation of phospholipase C (PLC) and protein kinase C (PKC) activation [36].

Besides the direct effects of Mg^2+^ on VSMCs, Mg^2+^ also modulates endothelial function, which in turn contributes to its vasodilatory actions. Normal endothelium plays a fundamental role in regulating vasomotor tone by synthesizing vasodilatory prostacyclin (PGI_2_) and nitric oxide (NO). Mg^2+^ has been shown to increase endothelial release of PGI_2_ in cultured human endothelial cells and in healthy human volunteers [17,36]. 

#### 2.2.2. MgD and Vascular Reactivity

Other Mg^2+^ effects could be due to altered binding of agonists to their specific cell membrane receptors and/or the production of vasoactive peptides, such as ATII and ET-1, which are powerful vasoconstrictors [38]. ATII, ET-1, vasopressin, and epinephrine/norepinephrine exert their vasoconstrictor effect via stimulation of AT1, ETA, V1a, and α_1_ receptors, respectively, on VSMCs. Activation of these Gq–protein-coupled receptors initiates the PLC, IP3, diacylglycerol, Ca^2+^, and PKC signal transduction pathway. Evidence suggests that following receptor–ligand interaction intracellular Mg^2+^ is also altered and that it too functions as a second messenger to modulate signal transduction [36]. For example, elevated levels of Mg^2+^ decrease ET-1 induced contraction, whereas decreased Mg^2+^ levels increase it [38]. Kharitonova et al. have explored the role of various Mg^2+^ compounds for the development of systemic inflammation and endothelial dysfunction in rats fed on a low Mg^2+^ diet for 74 days. Low Mg^2+^ diet reduces Mg^2+^ concentration in the plasma and red blood cells (RBCs), which is accompanied by a decreased concentration of endothelial NO synthase (eNOS), elevated levels of ET-1 in serum and impaired endothelial-dependent vasodilation. ET-1 produces multiple effects in the blood vessels: causes significant vasoconstriction, has proinflammatory effects, possesses mitogenic and proliferative properties, stimulates the formation of free radicals, and activates platelets. The analysis of the activity of the inorganic Mg^2+^ salts showed that supplementation with Mg-sulfate did not significantly reduce pathologically elevated levels of ET-1, but Mg-chloride completely restored the concentration of ET-1 to a normal value. Tested Mg-organic compounds Mg-oxybutyrate and Mg-*L*-aspartate reduce the concentration of ET-1 to a normal level, whereas treatment with Mg-*N*-acetyltaurate leads only to partial reduction of ET-1 in MgD groups [39]. Mg^2+^ is an essential cofactor of the enzyme δ-6-desaturase, which is the rate determining conversion of linoleic acid to gamma-linolenic, which in turn is converted to dihomo-γ-linoleic acid. The latter is a precursor of prostaglandin E_1_ (PGE_1_), which is both a vasodilator and inhibitor of platelet aggregation. MgD disrupts production of PGE_1_, which leads to vasoconstriction and increase in BP [17].

#### 2.2.3. MgD and the Renin-Angiotensin-Aldosterone System (RAAS)

Few studies have investigated the effects of MgD on the hormonal systems which control BP. The RAAS plays an essential role in humoral and hemodynamic regulation [40]. It was established, that in hypertensive patients with high plasma renin activity, serum Mg^2+^ was much lower than in normotensive persons [16]. MgD increases the ATII-induced plasma concentration of ALDO, as well as the production of thromboxane A_2_ and vasoconstrictor prostaglandins [3]. Mg^2+^ has some direct effects on ALDO synthesis rather than indirect effects via the RAAS. ALDO secretion from the zona glomerulosa of the adrenal gland is Ca^2+^ dependent process. Mg^2+^ infusion in humans decreases the production of ALDO, by inhibiting the cellular Ca^2+^ influx. MgD facilitates cellular Ca^2+^ influx, which may stimulate the production and release of ALDO [40]. It was proved that MgD rats have increased plasma renin activity and circulating levels of ALDO and corticosterone. Additionally, other authors indicate that Mg^2+^ supplementation decreases ATII stimulated production and release of ALDO from the adrenal cortex of normotensive subjects [16]. Rats fed with an MgD diet showed a continuous increment of the juxtaglomerular granulation index and width of the zona glomerulosa of the adrenal cortex, whereas the thickness of the inner zones diminished slightly. In Mg^2+^-recovering rats juxtaglomerular granulation index the width of the zona glomerulosa returned to normal [41]. Thus, one could suggest that MgD, by facilitating cellular Ca^2+^ entry, may promote ALDO production and release. Experimental studies have reported that MgD-induced HTN in rats is associated with increased vascular total Ca^2+^ content, and increased vasoconstrictor activity to endogenous agonists such as ATII and noradrenaline (NA) [40]. Mg^2+^ supplementation can reduce the pressor effect of ATII and stimulate the production of the vasodilator PGI_2_ [3].

#### 2.2.4. MgD and Catecholamines (CA)

Ca^2+^ exerts a major role in CA release from the adrenal gland and adrenergic nerve terminals in response to sympathetic stimulation. Because Mg^2+^ competes with Ca^2+^ for membrane channels, it can modify these types of Ca^2+^-mediated responses. The ability of Mg^2+^ to inhibit the release of CA from both the adrenal gland and peripheral adrenergic nerve terminals is well established in laboratory experiments. On the basis of these effects, Mg^2+^ can be used in patients where an excess of CA is prevalent, such as in phaeochromocytoma [42]. Acetylcholine (ACh) evoked CA release from adrenal glands. Ca^2+^ stimulates the secretion of ACh and there exists a direct relationship between Ca^2+^ concentration and the rate of CA release. Mg^2+^ antagonizes the stimulant effects of ACh on adrenal chromaffin cells; this effect can be overcome by the addition of Ca^2+^ [43]. Mg^2+^ is required for the catalytic action of adenylate cyclase (ADCY). For example, the decreased activity of the ADCY9 in the absence of Mg^2+^ results in increased secretion of ACh from preganglionic nerves, which in turn stimulates further release of CA from the adrenal glands [44]. On the other hand, Mg^2+^ together with ATP can greatly stimulate the release of CA from adrenal medullary granules. Neither ATP nor Mg^2+^ alone may have any effect on the release of CA. The effects of Ca^2+^, which cause the release of CA from the granules, are inhibited in the presence of ATP and Mg^2+^. The uptake of ^14^C-adrenaline (Adr) into the granules can also be stimulated by ATP and Mg^2+^ [45]. 

There are evidences that high concentrations of Mg^2+^ prevent the release of NA in some arteries by blocking *N*-type Ca^2+^ channels of nerve endings, which counteracts the rise in BP. Also rats fed with a MgD diet showed an increase in CA excretion [16]. The effects of Ca^2+^ and Mg^2+^ concentrations on responses to periarterial nervous sympathetic stimulation, NA, and tyramine have been investigated on the isolated rabbit central ear artery. An increase in Ca^2+^ concentrations potentiates responses to sympathetic stimulation until complete removal of Ca^2+^ inhibits these responses. The addition of the Mg^2+^ solution greatly reduced the responses to sympathetic stimulation, NA, and tyramine. These actions of Mg^2+^ on sympathetic transmission are important in determining the responsiveness of arterial smooth muscle to direct and indirectly acting sympathomimetic amines [46]. The effects of Adr infusion, sufficient to achieve its pathophysiological levels, and of therapeutic intravenous infusion of salbutamol, a β_2_-agonist, on plasma Mg^2+^ levels, were studied in a placebo-controlled design in healthy subjects. Plasma Mg^2+^ levels fell significantly during the Adr infusion and also during the salbutamol infusion, though more slowly. It is propose that intracellular shifts of Mg^2+^ occur as a result of β-adrenergic stimulation [47]. In another study, infusion of Adr in man significantly reduced the plasma Mg^2+^ levels in healthy males. This effect was abolished by simultaneous infusion of propranolol. NA had no such effect. These results suggest that the β-adrenergic system affect Mg^2+^ homeostasis [48]. On the other hand, dose-dependent increase in circulating Mg^2+^ was observed in rats infused with isoproterenol (ISO). Pretreatment with butoxamine or propranolol has prevented the ISO-induced increase in serum Mg^2+^ levels, whereas administration of atenolol has minimal effects. This evidence demonstrates the existence of a pool of Mg^2+^ that is mobilized into the circulation in response to selective β_2_-adrenergic stimulation [49].

### 2.3. MgD and Arterial Stiffness

Normally conduit arteries adapt pressure and blood flow during cardiac systole to facilitate perfusion to tissues during diastole. This is determined in large part by the elasticity, distensibility, and compliance of the arterial system. Loss of elasticity and increased stiffness demand greater force to accommodate blood flow, leading to increased systolic BP and consequent increased cardiac work load. Multiple interacting factors at the systemic (BP, hemodynamics), vascular (vascular contraction/dilatation, extracellular matrix remodeling), cellular (cytoskeletal organization and inflammatory responses), and molecular (oxidative stress, intracellular signaling, and mechanotransduction) levels contribute to arterial stiffness in HTN. Dysregulation of endothelial cells, VSMCs, and adaptive immune responses are also implicated in HTN [12]. Changes in Mg^2+^ concentrations play an important role in many of these processes.

#### 2.3.1. MgD, Low-Grade Inflammation, and Oxidative Stress at the Vascular Wall

A number of immunopathological mechanisms may be at the basis of HTN. There are evidence in animal models and humans that link HTN with changes in both humoral and cellular immunity, and in particular with the key role of low-grade vascular inflammation [50]. One study showed that the total intracellular Mg^2+^ is considerably lower in lymphocytes of the hypertensive patients, compared with healthy subjects, whereas serum Mg^2+^, erythrocyte Mg^2+^ and ionized platelet Mg^2+^ were not significantly different [51]. MgD leads to inflammation and increased production of free radicals [52] in the vascular wall, and they in turn contribute to the development of endothelial dysfunction and vascular remodeling. Low Mg^2+^ intake is associated with a higher probability of increased serum C-reactive protein (CRP) levels in children [53]. There is also an association between the dietary intake of Mg^2+^ and elevated CRP levels in the adult population. Insufficient dietary intake of Mg^2+^ may be associated with an increased inflammatory response resulting in more frequent occurrence of cardiovascular accidents [54]. Intake of Mg^2+^ is also inversely related to the level of hs-CRP, interleukin 6 (IL-6), and fibrinogen [55]. MgD significantly increases production of various proinflammatory molecules such as, interleukin 1 (IL-1), IL-6, tumor necrosis factor α (TNF-α), vascular cell adhesion molecule-1 (VCAM), plasminogen activator inhibitor-1 (PAI-1), and decreases expression and activity of the antioxidant enzymes such as glutathione peroxidase, superoxide dismutase, and catalase. Cellular and tissue levels of important antioxidants such as glutathione, vitamin C, vitamin E and selenium are also reduced [16]. This shows that MgD can increase cytotoxicity of the free radicals to endothelial cells [56]. For example Mg^2+^ deficit, in rats leads to an increase in the inflammatory mediators, such as histamine, IL-1, IL-6, TNF-α, and ET-1, which is associated with leukocytosis and generation of free radicals [34] (Figure 1).

#### 2.3.2. MgD, Vascular Structure and Remodeling

Vascular remodeling is a permanent process of structural changes in the vessel wall in response to a number of hemodynamic stimulus [57]. In HTN, resistance arteries undergo an inward remodeling, while larger arteries show outward hypertrophic remodeling [58,59,60]. The vascular extracellular matrix (ECM) comprises multiple structural proteins, including collagens, elastin, fibronectin, and proteoglycans. The absolute and relative quantities of collagen and elastin determine biomechanical properties of vessels. Excessive ECM protein deposition, in particular collagen and fibronectin, contributes to increased intima–media thickening, vascular fibrosis, and stiffening leading to the development of HTN [12]. Mg^2+^ regulates collagen and elastin turnover and the structure of the ECM. MgD leads to a delay in the synthesis of all structural molecules (collagen, elastin, proteoglycans and glycosaminoglycans) (Figure 1). Hyaluronan synthases (HAS)—HAS1, HAS2 and HAS3, contain Mg^2+^ ion in their active centers. On the other hand it is known that inhibitors of the hyaluronidase (HYAL) depend on the concentration of Mg^2+^ ions. Thus, low levels of Mg^2+^ could lead to decreased activity of HAS, and at the same time to an increased activity of HYAL. Tissue transglutaminase (TG2) is an enzyme of the transglutaminase superfamily that is ubiquitously expressed in the vasculature [61]. TG2 is associated with a wide range of CVD and processes, including the development of HTN, and the progression of atherosclerosis, regulating vascular permeability, and angiogenesis. TG2 activity is associated with arterial stiffening in humans and rats. TG2 forms glutamine-lysine cross-links between variety of extracellular proteins, including collagen and elastin [62]. TG2 is secreted through a Golgi-independent mechanism to the ECM, where it can be activated to a Ca^2+^-bound open conformation to catalyze the formation of isopeptide bonds [61]. Thus, Ca^2+^ may be limiting TG2 activity in the ECM [62]. TG2 is activated by Ca^2+^, and inhibited by Mg^2+^ [63]. A disturbance in lysyl oxidase (LOX) expression has also been reported in CVD, and an increase in vascular LOX activity has been described in experimental models of HTN. Mg^2+^ can inhibit LOX, which is also associated with crosslinking of chains of elastin and/or collagen [63,64]. Additionally, MgD could lead to the production of defective collagen, elastin, and fibronectin by fibroblasts [65].

Fundamental to many of the processes underlying ECM reorganization and fibrosis in HTN is activation of matrix metalloproteinases (MMPs) and tissue inhibitors of metalloproteinases (TIMPs). ECM MMPs and TIMPs may contribute to the profibrotic phenotype in HTN. Activated MMPs degrade collagen, elastin, and other ECM proteins, resulting in a modified ECM, often associated with a proinflammatory microenvironment that triggers a shift of endothelial and VSMCs to a more secretory, migratory, and proliferative phenotype, which contributes to fibrosis, calcification, endothelial dysfunction, and increased intima–media thickness, further impacting on vascular remodeling and arterial stiffness [12]. In endothelial cells cultured in MgD medium, a significant increase in expression and activity of MMP-2 and MMP-9 has been reported. Also, MMP-2 and MMP-9 have been associated with alterations of the vascular wall in Mg^2+^-deficient rats [66]. In addition, there is evidence that the addition of Mg-sulfate effectively attenuated MMP-9 activity in а human umbilical cord vein endothelial cell line [67]. These data are confirmed by K. Kostov et al. who find that in patients with essential HTN there was a moderate negative correlation of serum Mg^2+^ with MMP-2 (r = −0.318, *p* = 0.013). There was a similar correlation of Mg^2+^ with MMP-9 in patients with HTN and T2D (r = −0.376, *p* = 0.003). The results show that lower and higher serum Mg^2+^ levels correlate inversely with MMP-2 and MMP-9 levels in HTN [68]. It is noteworthy that in Mg^2+^-deficient endothelial cells, MMP-2 and MMP-9 activity overrides the inhibitory effect of TIMP-2, which probably is induced as an attempt to counterbalance the effects of the proteases [66]. A nuclear factor (NF)-κB-binding site is present in the promoter of the MMP-9 gene. It is therefore possible that low Mg^2+^ availability might directly increase MMP-9 expression via NF-κB [66,69]. In cultured rat VSMCs, Mg^2+^ significantly reduced the production of MMP-2 under basal and platelet-derived growth factor-stimulated conditions in a dose-dependent manner, while neither verapamil nor nifedipine showed any effect under the same conditions. These data suggest that the beneficial effect of Mg^2+^ supplementation on vascular disease processes may be due, at least in part, to the inhibitory effect of Mg^2+^ on the production of MMP-2 in VSMCs [70]. Evidence supporting this data is that in cultured rat cardiac fibroblasts, Mg^2+^ significantly reduced the production of MMP-2 in a dose-dependent manner [71]. MgD may increase the activity of MMPs, including collagenases, which begin to degrade the extracellular vascular matrix and primarily collagen with an increased speed. The degradation of elastin fibers can significantly increase (up to 2–3 times) in the presence of Mg^2+^. MgD is associated with low elastase activity and an increased number of elastic fibers [63]. Altura et al. describe and other possible mechanisms by which MgD can affect vascular remodeling processes. They present new evidence for effects on platelet-activating factor, proto-oncogenes, and sphingolipids, e.g., ceramide and sphingosine with upstream regulation in both VSMCs and cardiac muscle cells. These findings will be helpful in explaining many of the known cardiovascular manifestations of MgD, especially vascular remodeling seen in atherosclerosis and HTN [72].

#### 2.3.3. MgD, Endothelial Dysfunction and Atherosclerosis 

MgD may potentiate the development of endothelial dysfunction via activation of NF-κB, which includes the transcriptional program leading to development of the proinflammatory phenotype [69]. Low extracellular Mg^2+^ slows endothelial cell proliferation, stimulates the adhesion of monocytes, and affect the synthesis of vasoactive molecules, such as NO and PGI_2_. Endothelial function is significantly impaired in a model of familial hypomagnesemia in mice. Compared to controls, in the aortas of these animals were found reduced amounts of eNOS and increased expression of proinflammatory molecules, such as VCAM, PAI-1, as well as of the TRPM7 channel [19]. Endothelial dysfunction is an early event in the process of atherogenesis and precedes the angiographic and ultrasound evidences of damage to the arterial wall [66]. The pathogenesis of atherosclerotic changes and disturbances in endothelial function are complex and multifactorial. In this context, Mg^2+^ deficit is too important [73]. This mineral is especially important because of its antiatherosclerotic effects [74]. Endothelial function correlates to the levels of Mg^2+^ and results of Mg^2+^ supplementation have showed significantly improved endothelial function in patients with ischemic heart disease and diabetes. These results in humans have also been observed in different experimental models in which Mg^2+^ deficit affects vascular structure and function. Low levels of extracellular Mg^2+^ favor and increase endothelial permeability. More specifically, MgD enhance the transport of low-density lipoproteins (LDL) through the endothelial layer [66]. Several studies have reported beneficial effects of Mg^2+^ supplementation on plasma LDL levels, as well as on high-density lipoproteins (HDL) levels, which are increased [75]. Another possibility by which Mg^2+^ contributes to the development of atherogenesis is through the effect on triglyceride levels which are increased in MgD. Accumulation of triglyceride-rich lipoproteins is accompanied by decreased concentration of HDL and increased plasma concentration of apolipoprotein B. Since the oxidation of lipoproteins play a key role in the development of atherosclerosis, it could be another mechanism by which Mg^2+^ influences. It is also possible proatherogenic lipoprotein changes found in MgD to be a consequence of the inflammatory response [76]. A central role for Mg^2+^ mediated effects on endothelial cells has IL-1α, which is regulated by NF-κB and may be inducer of the NF-κB. IL-1α increases significantly in the environment of low Mg^2+^ content. IL-1α also induces the production of various chemokines and adhesion molecules in vascular endothelial cells by activation of NF-κB, and thus favors aggregation, adhesion, and diapedesis of monocytes. In particular, low concentrations of Mg^2+^ stimulate the secretion of interleukin 8 (IL-8), and chemokines, which are overexpressed in human atherosclerotic lesions. IL-8 is essential for chemotaxis and adhesion of monocytes to endothelial cells, which is a fundamental event in the initiation of atherogenesis and also stimulates proliferation and migration of VSMCs. By induction of IL-1α, low serum Mg^2+^ may also stimulate overexpression of VCAM-1 on the surface of endothelial cells which assists in the migration of leukocytes. In addition, the secretion of granulocyte-macrophage colony-stimulating factor is significantly higher in endothelial cells with Mg^2+^ deficit [66]. All these date indicate that the MgD is a potential factor for accumulation of monocytes/macrophages in the arterial wall during the early stages of atherosclerosis (Figure 1).

#### 2.3.4. MgD, MetS, and T2D

The presence of MetS is also associated with altered Mg^2+^ metabolism [77]. Usually, the triad consisting of obesity, HTN, and impaired glucose tolerance/insulin resistance is denoted as MetS [78]. Furthermore, HTN is present in a high proportion of patients with T2D [79,80]. A common feature in patients with T2D, HTN, and low levels of HDL is MgD (Table 1). 

Currently, there is little data on serum Mg^2+^ levels in people with MetS. The relationship between the intake of Mg^2+^ and MetS was investigated prospectively in 5115 young Americans (aged 18–30 years), initially without MetS and diabetes, which were enrolled in Coronary Artery Risk Development in Young Adults (CARDIA) study from 1985 to 1986. The total number of participants included in the analysis was 4637, and 74% were evaluated at the 15-year period in 2000–2001. During this interval 608 cases of MetS were diagnosed. The findings showed that young people with a high Mg^2+^ intake had a lower risk of developing MetS and that risk was dose-dependent [78]. Guerrero-Romero et al. found a link between Mg^2+^ levels, inflammation, and oxidative stress, as risk factors for the development of MetS. Mg^2+^ intake is inversely proportional to the components of MetS and fasting insulin levels, suggesting that higher Mg^2+^ intake may have a protective role against the risk of developing MetS [77]. The results of several clinical studies have shown that increased synthesis and release of proinflammatory cytokines trigger the process of chronic inflammation, which may be the link between obesity and MetS. On the other hand hypomagnesemia triggers low-grade chronic inflammation and Mg^2+^ deficit may be associated with the development of MetS. These findings support the hypothesis that MgD can play an important role in the pathophysiology of MetS, and the actuation of the inflammatory reaction caused by the shortage of Mg^2+^ could be the link between MgD and MetS [81]. Some studies linked decreased Mg^2+^ levels with chronic inflammatory stress in obese people. Obesity affects over 35% of the adult population of the USA and is a main risk factor for chronic diseases, associated with a lower Mg^2+^ status, such as atherosclerosis and T2D. MgD is often found in people with MetS and T2D, which are connected with higher plasma concentrations of CRP [82]. 

Mg^2+^ plays a very important role in the development of T2D [83]. Corica et al. have recently shown that patients with T2D having lipid profile with high risk, high BP, and abdominal obesity, have lower levels of Mg^2+^, compared with patients without metabolic risk factors. Furthermore, plasma triglycerides and waist circumference were independently associated with hypomagnesaemia [77]. T2D is often linked with hypomagnesaemia, as has been reported at an occurrence rate of 13.5–47.7% [78]. The relationship between insulin and Mg^2+^ is bipartite. Insulin regulates Mg^2+^ homeostasis, but on the other hand Mg^2+^ is a major factor determining insulin and glucose metabolism. Extracellular Mg^2+^ acts as Ca^2+^ antagonist and inhibits Ca^2+^ influx, required for insulin secretion. Thus a decreased concentration of extracellular free Mg^2+^ results in an increased Ca^2+^ influx and increased concentration of intracellular free Ca^2+^. The increased intracellular Ca^2+^ stimulates insulin secretion by beta-cells, as was demonstrated in experiments with an insulinoma cell line [84]. The effect of extracellular Mg^2+^ on insulin secretion was found in healthy human subjects. In subjects with 0.79 mmol/L plasma Mg^2+^, fasting plasma insulin was 23 μU/mL, while in those with plasma Mg^2+^ 0.87 or 1.00 mmol/L, fasting plasma insulin amounted to 11 μU/mL [85]. 

There are growing evidences that highlight the clinical significance of altered Mg^2+^ metabolism for the occurrence of peripheral insulin resistance. MgD can lead to disturbances of the tyrosine kinase activity of the insulin receptor (IR), associated with the development of post receptor insulin resistance and reduced cellular glucose utilization, as a lower Mg^2+^ concentration, requires a greater amount of insulin for glucose metabolism [77]. The effects of MgD on glucose-stimulated insulin secretion and insulin action on skeletal muscle were studied in experimental animals. The hypothesis that changes in Mg^2+^ metabolism induce insulin resistance is confirmed by data showing that lower dietary intake of Mg^2+^ is associated with insulin resistance. Rats fed on a low Mg^2+^ diet showed a significant increase of blood glucose and triglycerides. The insulin resistance, observed in the skeletal muscle of rats with MgD is partially associated with a defect of tyrosine kinase activity of the IR [86,87,88]. Insulin action begins with the binding of insulin to an IR on the cell membrane of the target cells. The IR is a transmembrane glycoprotein with tyrosine kinase activity [89]. Activation of the receptor is an important step in transmembrane signaling for insulin action. The activated kinase promotes autophosphorylation of receptor tyrosine residues. The insulin–receptor complex is internalized and phosphorylates IR substrates 1–6 (IRS 1–6) and other kinases in the insulin signaling cascade [90]. When the intrinsic tyrosine kinase activity of the receptor is triggered by insulin binding, two major signaling pathways have been activated: (1) Ras-mitogen-activated protein kinase (MAPK) pathway, which controls cell growth and differentiation; (2) Phosphoinositide 3-kinase/Akt (PI3K/Akt) pathway. Binding of IRS to the regulatory subunit of phosphoinositide 3-kinase (PI3K) results in activation of PI3K, which phosphorylates membrane phospholipids and phosphatidylinositol 4,5-bisphosphate (PIP2). This complex activates the 3-phosphoinositide-dependent protein kinases (PDK-1 and PDK-2) resulting in activation of Akt/protein kinase B and atypical protein kinase [91,92]. Activated Akt phosphorylates its 160 kDa substrate, which stimulates the translocation of insulin-mediated glucose transporter type 4 (GLUT4) from intracellular vesicles to the plasma membrane [93]. The PI3K/Akt pathway is a key component of the insulin signaling cascade, which is necessary for the metabolic effects of insulin and GLUT4 translocation [94]. Since Mg^2+^ is a necessary cofactor in all ATP transfer reactions, intracellular Mg^2+^ concentration is critical in the phosphorylation of the IR and other kinases [95]. In all these reactions Mg^2+^ operates together with ATP as a kinase substrate. Additionally Mg^2+^ is bound to a regulatory site of the IR tyrosine kinase (IRTK). The apparent affinity of the IRTK for Mg^2+^-ATP increased as the concentration of free Mg^2+^ increased, and the apparent affinity of the IRTK for free Mg^2+^ increased as the concentration of Mg^2+^-ATP increased [84]. There are evidences that show a link between decreased Mg^2+^ concentration and reduction of tyrosine-kinase activity at the IR level, which results in the impairment of insulin action and development of insulin resistance [96]. Studies in multiple insulin resistant cell models have demonstrated that an impaired response of the tyrosine kinase to insulin stimulation is one potential mechanism causing insulin-resistant state in T2D [97]. Nadler et al. have reported that insulin sensitivity decreases even in nondiabetic individuals after induction of MgD [98]. Finally, Mg^2+^ can also be a limiting factor in carbohydrate metabolism, since many of the enzymes in this process require Mg^2+^ as a cofactor during reactions that utilize the phosphorus bond [99].

Inadequate dietary intake of Mg^2+^ is an independent risk factor for the development of T2D. Lopez-Ridaura et al., evaluating 37,309 participants free of cardiovascular disease and T2D, found a significant inverse association between Mg^2+^ intake and diabetes risk [100]. Van Dam et al. reported a similar relationship. Their findings indicated that a diet high in Mg^2+^-rich foods, particularly whole grains, is associated with a substantially lower risk of T2D [101]. Benefits of Mg^2+^ supplementation in diabetic subjects have been found in some clinical studies. Rodriguez-Moran et al. reported that Mg^2+^ supplementation improves insulin sensitivity and secretion as well as metabolic control in patients with T2D [96]. Mooren et al. have shown beneficial effect of oral Mg^2+^ supplementation on insulin sensitivity in overweight, nondiabetic subjects [102].

#### 2.3.5. MgD and Vascular Calcification

Vascular calcification is the extracellular deposition of Ca^2+^ in the arterial wall and is intimately linked with the HTN. On the other hand, HTN was considered a risk factor for atherosclerosis and associated calcification. Two types of extracellular vascular calcification are recognized, intimal and medial. Intimal calcification is exclusively associated with atherosclerosis. Medial calcification may contribute to increasing BP by decreasing the elasticity of the media. Decreased elasticity results in arterial stiffening which accelerates pulse wave velocity and widening the pulse pressure, leading ultimately to HTN. Intimal and, especially, medial vascular calcification are associated with arterial stiffening, the major cause of isolated systolic HTN in the elderly [103]. The first in vitro evidence in human aortic VSMCs for a protective role of Mg^2+^ on vascular calcification was based on the observation that living cells are necessary for Mg^2+^ ions to exert its protective effect. These studies suggested a potentially active intracellular role for Mg^2+^ ions in attenuating the vascular calcification process [33,35,104]. In confirmation of this, Hruby et al. reported on favorable associations between dietary and supplemental Mg^2+^ intake and lower calcification of the coronary arteries [105]. Furthermore, it was found that higher Mg^2+^ levels prevented calcification of bovine VSMCs, and further progression of the already established calcification. Inhibition of the Wnt/β -catenin signaling pathway was identified as one of the possible intracellular mechanisms by which Mg^2+^ achieved its anti-calcifying effect [104].

#### 2.3.6. MgD and Vascular Aging

Aging represents a major risk factor for MgD. The total body Mg^2+^ content and intracellular Mg^2+^ tend to decrease with age. Aging is often associated with a Mg^2+^ deficiency due to reduced intake and/or absorption, increased renal wasting and/or reduced tubular reabsorption, as well as age-related illnesses and their treatment with certain drugs [106]. The aging process is associated with alterations in the properties of all the elements of the vascular wall, including endothelium, VSMCs, and ECM. This increases vascular stiffness and leads to the development of isolated systolic HTN. “Aging”-associated arterial changes and those associated with HTN (and early atherosclerosis and diabetes) are fundamentally intertwined at the cellular and molecular levels [107]. At the molecular and cellular levels, arterial aging and HTN-associated vascular changes are characterized by reduced NO production, increased generation of reactive oxygen species (oxidative stress), activation of transcription factors, induction of “aging” genes, stimulation of proinflammatory and profibrotic signaling pathways, reduced collagen turnover, calcification, VSMCs proliferation, and ECM remodeling. These processes contribute to increased fibrosis, which is further promoted by prohypertensive vasoactive agents, such as ATII, ET-1, and ALDO [12]. The cellular and molecular proinflammatory mechanisms that underlie arterial aging are novel putative candidates to be targeted by interventions aimed at attenuating arterial aging, and thus possibly attenuating the major risk factors for HTN and atherosclerosis [107]. Targeted interventions aimed at correcting MgD and maintaining an optimal Mg^2+^ balance may prove to be an appropriate strategy against arterial aging due to its positive effects on low-grade inflammation and oxidative stress associated with aging process (Figure 1).

### 2.4. MgD and Stress Response

Stress is among the potential psychological risk factors for HTN. Acute stressful events have no consistent association with the HTN. Chronic stress on the other hand, particularly the non-adaptive response to stress, may be a more likely cause of sustained elevation of BP. The mechanisms underlying the association between psychosocial stress and HTN can be divided into behavioral, psychological and pathophysiological. The latter involves neuro-endocrine activation mediated by the hypothalamic pituitary adrenal axis (HPAA) [108]. Mg^2+^ plays a key role in the activity of psychoneuroendocrine systems. For example, all elements of the limbic-HPAA are sensitive to the action of Mg^2+^. Mg^2+^ modulates activity of the HPAA which is a central substrate of the stress response system. Activation of the HPAA instigates adaptive autonomic, neuroendocrine, and behavioral responses to cope with the demands of the stressor [10]. MgD induced an increase in the transcription of the corticotropin releasing hormone in the paraventricular hypothalamic nucleus, and elevated adrenocorticotropic hormone (ACTH) plasma levels, indicating an enhanced set-point of the HPAA [109]. MgD results in a stressor effect and increases susceptibility to the physiological damage produced by stress (Figure 1). Mg^2+^ supply has been shown to attenuate the development of adverse stress reactions. Stress activates the HPAA and the sympathetic nervous system. The innervation of the kidney may result in the overproduction of renin, which in turn activates the production of ATII, a powerful vasoconstrictor that elevates the BP [110]. Additionally, Mg^2+^ deficient mice are more sensitive to anxiety-provoking situations. Dysregulation of the HPAA evoked by MgD is normalizes by chronic desipramine or diazepam treatment. These data indicate that dysregulation in the HPAA may contribute to hyper-emotionality in response to dietary induced hypomagnesaemia [109].

Mg^2+^ ions also have a key role in the modulation of neurotransmission. Numerous studies have confirmed that the function of the native *N*-methyl-d-aspartate (NMDA) receptor is the result of equilibrium between extracellular and intracellular concentration of Mg^2+^. The blockade of the ion channel of the NMDA receptor is the most well-known and established way in which Mg^2+^ affects the functioning of the central nervous system (CNS) [109]. Mg^2+^ reduces neuronal hyperexcitability by inhibiting NMDA receptor activity and also is essential for the activity of metabotropic glutamate receptors (mGluRs) in the brain. The mGluRs play a key modulatory role in glutamatergic activity, secretion, and presynaptic release of glutamate, activity of the gamma-aminobutyric acid (GABA)-ergic system, and regulation of the neuroendocrine system. Mg^2+^ may additionally modulate anxiety via increasing GABA availability by decreasing presynaptic glutamate release. GABA is a primary inhibitory transmitter in the CNS that counterbalances the excitatory action of glutamate [111]. The state of acute and chronic stress leads to depletion of intracellular Mg^2+^ and its loss in the urine, because in stressful situations secreted elevated amounts of Adr and NA help to remove Mg^2+^ from the cells. Intracellular RBCs Mg^2+^ depletion is found in patients with HTN. MgD affects the balance of monoamines, such as CA and serotonin in the brain. CA released into the blood is rapidly inactivated by the enzyme catechol-*O*-methyl transferase (COMT). The latter is activated by Mg^2+^ and is inhibited by Ca^2+^. MgD leads to decreased activity of COMT, which in turn increases the concentration of circulating CA [44]. Brain NA was determined in adult male mice with genetically low (MGL) or high (MGH) blood Mg^2+^ levels. NA levels were significantly higher in MGL than in MGH mice. These data together with the higher urinary NA excretion observed in the MGL line might account for the higher sensitivity and/or reactivity of MGL animals to stress [112]. Moreover, in stressful conditions, MGL mice displayed a more aggressive behavior than the control MGH strain. Altogether, MGL mice showed a more restless behavior, and much higher brain and urine NA levels than the MGH animals [113]. An analysis of the literature suggests the possible role of MgD in the susceptibility to CVD, observed among subjects displaying a type A behavior pattern. Type A subjects are more sensitive to stress and produce more CA than type B subjects. This, in turn, seems to induce an intracellular Mg^2+^ loss. In the long run, type A individuals would develop a state of MgD, which may promote a greater sensitivity to stress, and ultimately to the development of CVD [114], including HTN. Hypomagnesemia usually involves cellular Mg^2+^ depletion, but acute stress that increase serum concentrations of CA may lower serum Mg^2+^ concentration, which does not always imply depleted tissue Mg^2+^ stores [115]. 

The potential effect of Mg^2+^ in attenuating psychological response to stress merits further investigation since stress is a ubiquitous feature of modern life. The modulation of HPAA by Mg^2+^, which has been shown to reduce central (ACTH), peripheral (cortisol) endocrine responses [111], and reduces neuronal hyperexcitability by NMDA, mGluRs and GABA-effects suggests that behavioral responses to stress exposure may be attenuated by Mg^2+^ supplementation in patients with HTN.

## 3. MgD, Groups at Risk, Replacement Therapy and Prevention

### 3.1. Mg^2+^ Supplements in Hypertensive Subjects

HTN is a complex, heterogeneous disorder whose etiology, pathogenesis, and treatment still raises some unresolved questions. Maintenance of optimal Mg^2+^ status in the human body may help prevent or treat HTN. Although most epidemiological and experimental studies support a role of MgD in the pathophysiology of HTN, data from clinical studies have been less convincing [38]. In some studies the inverse association between Mg^2+^ and BP remained inconclusive, but not in others [116]. Ultimately, the view is that MgD in patients with HTN is linking with significant adverse effects on BP. In the Atherosclerosis Risk in Communities (ARIC) study, serum Mg^2+^ level in hypertensive patients was inversely proportional to the systolic BP. The study examined a cohort of 15248 participants aged 45–64 years [117]. In another meta-analysis of 34 randomized, double-blind, placebo-controlled trials involving a total of 2028 subjects, it was found that oral administration of Mg^2+^ resulted in a significant reduction in both systolic and diastolic BP (2.00 mmHg and 1.78 mmHg respectively) [118]. An analytical review of 44 studies in humans have shown that low doses of Mg^2+^ supplementation, for example 243 mg/day can significantly lower BP in patients with uncomplicated HTN, treated six months or longer with antihypertensive drugs [119]. Moreover, the researchers reported that patients with MgD require higher doses of antihypertensive drugs compared to those with normal Mg^2+^ concentration [118]. The evidence supporting the cause–consequence antihypertensive effect of Mg^2+^ in adults suggest that oral Mg^2+^ supplements may be recommended for the prevention of arterial HTN or as adjuvant antihypertensive therapy [11]. It should be noted that MgD is not found in all patients with HTN. On the other hand, not all people with hypomagnesemia have high BP. These differences are probably due to the fact that patients with high BP do not constitute a homogenous group [78]. This may be one of the possible causes for the discrepancy between epidemiological and clinical data. Despite these discrepancies concerning Mg^2+^ status and high BP, some hypertensive patients constantly demonstrate hypomagnesemia. Among them are patients with obesity, insulin resistance, hypertriglyceridemia, severe forms of HTN, hyperaldosteronism (volume-dependent HTN), pregnancy induced HTN, and patients of African-American origin [120]. In view of the still ill-defined role of Mg^2+^ in clinical HTN, Mg^2+^ supplementation is advised in those hypertensive patients who are receiving diuretics, who have resistant or secondary HTN or who have frank MgD [121]. In the USA, the Estimated Average Requirement (EAR) and RDA of Mg^2+^ for adult women are set at 255–265 mg and 310–320 mg/day, respectively. The EAR and RDA of Mg^2+^ for adult men are set at 330–350 mg and 410–420 mg/day, respectively. These dietary reference intakes are based on data from 16 men and 18 women who have consumed self-selected diets and have had a decreased Mg^2+^ intake during the balance periods, which could have affected balance values [122,123]. The current RDA for Mg^2+^ ranges from 80 mg/day for children 1–3 years of age to 130 mg/day for children 4–8 years of age. For older males, the RDA for Mg^2+^ ranges from as low as 240 mg/day (range, 9–13 years of age) and increases to 420 mg/day for males 31–70 years of age and older. For females, the RDA ranges from 240 mg/day (9–13 years of age) to 360 mg/day for females 14–18 years of age. The RDA for females 31–70 years of age and older is 320 mg/day. Many nutritional experts feel the ideal intake for Mg^2+^ should be based on the body weight (e.g., 4–6 mg per kg/day) [4]. Intravenous Mg^2+^ supplementation may be more effective, but this treatment has the disadvantage that it requires regular hospital visits. The treatment regimen of intravenous Mg^2+^ supplementation normally consists of 8–12 g of Mg-sulfate in the first 24 h followed by 4–6 g/day for 3 or 4 days. When serum Mg^2+^ levels are extremely low or are accompanied by hypokalemia, Mg^2+^ supplementation may not be sufficient to restore normal Mg^2+^ levels. In that case, patients are often cosupplemented with K^+^ or receive amiloride to prevent K^+^ secretion [124]. Recent reports indicate that individuals with serum Mg^2+^ concentrations >0.75 mmol/L, or high as 0.85 mmol/L, could be Mg^2+^-deficient. Thus, to assess Mg^2+^ status of an individual with a serum Mg^2+^ concentration between 0.75 and 0.85 mmol/L is requires additional measures of status. A urinary excretion of <80 mg (3.29 mmol)/day and/or dietary intake history showing a Mg^2+^ intake of <250 mg/day would support the presence of MgD [122]. In the treatment of MgD are recommended organic bound Mg^2+^ salts, such as Mg^2+^ citrate, gluconate, orotate, or aspartate, because of their high bioavailability [4,125].

Hypermagnesemia is a rare condition and is seen most often in patients with renal impairment who take medicines containing Mg^2+^. Excessive intake of supplemental Mg^2+^ can result in adverse effects, especially in impaired renal function. Serum concentrations >8 mmol/L cause drowsiness, vasodilation, slowing of atrioventricular conduction, and hypotension [126,127,128].

### 3.2. Food and Water Sources of Mg^2+^

#### 3.2.1. Mg^2+^ Intake from Food

In the Western World, dietary intake of Mg^2+^ is subnormal, with shortfalls of between 65 and 225 mg of Mg^2+^/day, depending upon geographic region. Several epidemiologic studies in North America and Europe have shown that children and adults, some which are pregnant women, consuming Western-type diets are low in Mg^2+^ content (i.e., 30–50% of the RDA for these populations) [129]. Epidemiological observations suggest a negative correlation between dietary Mg^2+^ intake and BP [11]. Overall, the current evidence supports the importance of adequate dietary Mg^2+^ intake for the reduction of BP and total CVD risk. These findings support the importance of increasing the consumption of Mg^2+^-rich foods, including fruits, vegetables, nuts, and whole grains in the treatment and prevention of high BP [125]. A Mg^2+^-rich diet should be encouraged in hypertensive subjects as well as in predisposed communities because of the advantages of such a diet in the prevention of HTN [121]. The Dietary Approaches to Stop HTN (DASH) diet (originally termed the “combination diet”4) contains larger amounts of Mg^2+^, K^+^, Ca^2+^, dietary fiber, and protein and smaller amounts of total and saturated fat and cholesterol than the typical diet [130]. The Mediterranean diet is also rich in Mg^2+^, dietary fiber, antioxidant capacity, and polyphenolic compounds [131]. In trials of vegetarian diets, replacing animal products with vegetable products reduced BP in normotensive and hypertensive people. Aspects of vegetarian diets believed to reduce BP include their high levels of fiber and minerals (such as Mg^2+^ and K^+^) and their reduced fat content. In observational studies, significant inverse associations of BP with intake of Mg^2+^, K^+^, Ca^2+^, and fiber have also been reported [132].

#### 3.2.2. Mg^2+^ Intake from Water

Water is a variable source of Mg^2+^ intake. Typically, water with increased “hardness” has a higher concentration of Mg^2+^ salts. Since this varies depending on the area from which water comes, Mg^2+^ intake from water is usually not estimated to a sufficient extent. This omission may lead to impaired assessment and underestimation of total intake of Mg^2+^ in certain regions [123]. The modern processed food diet, which is low in Mg^2+^ and is spreading globally, makes this well-researched potential of drinking-water Mg^2+^ worth serious consideration, especially in areas where insufficient dietary intake of Mg^2+^ is prevalent. It would be wise and forward-thinking for public health officials to consider how high-Mg^2+^ drinking water might be made available to communities, i.e., water with Mg^2+^ levels of at least 10 mg/L and ideally 25–100 mg/L [133]. 

## 4. Conclusions

The enhancing effect of MgD on BP should be considered in the context of total intake and loss of Mg^2+^ in each individual patient with HTN. Special attention should be given to the risk groups in which serum Mg^2+^ levels should be monitored periodically. Considering the numerous positive effects of Mg^2+^ on a number of mechanisms related to HTN, consuming a healthy diet that provides the recommended amount of Mg^2+^ can be an appropriate strategy for helping control BP.

## Figures and Tables

**Table 1:** Negative effects of MgD on the body and organs.

**General**: anxiety, agitation, irritability, headache, loss of appetite, nausea.
**Musculature:** muscle spasm and tetany.
**CNS/Nerves:** nervousness, migraine, depression, poor memory, low stress tolerance, paraesthesia, tremor, and seizures.
**Cardiovascular system:** HTN, risk of arrhythmias, coronary spasm, atherosclerosis, endothelial dysfunction, low-grade vascular inflammation, arterial stiffness, vascular ECM remodeling, arterial calcification, vascular aging, increased platelet aggregation, potentiates Ca^2+^-mediated vasoconstriction, potentiates the vasoconstrictor effects of ATII, ET-1, NA, Adr, and TxA_2_.
**Electrolytes:** sodium retention, hypokalemia, and hypocalcemia.
**Metabolism:** dyslipoproteinemia, insulin resistance, pancreatic β-cell dysfunction, decreased glucose tolerance, increased risk of MetS and T2D, disorders of vitamin D metabolism, resistance to PTH, and osteoporosis.
**Pregnancy:** pregnancy complications (e.g., eclampsia).
**Gastrointestinal tract:** constipation.

## Abbreviations

MgD	Magnesium deficiency
Mg^2+^	Magnesium
Ca^2+^	Calcium
K^+^	Potassium
Na^+^	Sodium
HTN	Hypertension
MetS	Metabolic syndrome
T2D	Type 2 diabetes
RDA	Recommended Dietary Allowance
EAR	Estimated Average Requirement
CVD	Cardiovascular diseases
BP	Blood pressure
WHO	World Health Organization
TRPM6	Transient receptor potential melastatin-6 channel
TRPM7	Transient receptor potential melastatin-7 channel
VSMCs	Vascular smooth muscle cells
ET-1	Endothelin-1
ATII	Angiotensin II
ALDO	Aldosterone
SHR	Spontaneously hypertensive rats
WKY	Wistar-Kyoto rats
EGF	Epidermal growth factor
PTH	Parathyroid hormone
IP3	Inositol-1,4,5-trisphosphate
PIP2	Phosphatidylinositol 4,5-bisphosphate
AT1	Angiotensin II receptor type 1
ETA	Endothelin A receptor
V1a	Vasopressin receptor 1a
PLC	Phospholipase C
PKC	Protein kinase C
PGI_2_	Prostacyclin
NO	Nitric oxide
RBCs	Red blood cells
eNOS	Endothelial nitric oxide synthase
PGE_1_	Prostaglandin E_1_
RAAS	Renin-Angiotensin-Aldosterone System
NA	Noradrenaline
Adr	Adrenaline
CA	Catecholamines
ACh	Acetylcholine
ADCY	Adenylate cyclase
ISO	Isoproterenol
CRP	C-reactive protein
IL	Interleukin
TNF-α	Tumor necrosis factor-α
VCAM	Vascular cell adhesion molecule-1
PAI-1	Plasminogen activator inhibitor-1
ECM	Extracellular matrix
HAS	Hyaluronan synthases
HYAL	Hyaluronidase
TG2	Transglutaminase
LOX	Lysyl oxidase
MMPs	Matrix metalloproteinases
TIMPs	Tissue inhibitors of metalloproteinases
NF-κB	Nuclear factor kappa B
LDL	Low-density lipoproteins
HDL	High-density lipoproteins
IR	Insulin receptor
MAPK	Mitogen-activated protein kinase
PI3K	Phosphoinositide 3-kinase
PIP2	Phosphatidylinositol 4,5-bisphosphate
GLUT4	Glucose transporter type 4
IRTK	Insulin receptor tyrosine kinase
CNS	Central nervous system
HPAA	Hypothalamic Pituitary Adrenal Axis
ACTH	Adrenocorticotropic hormone
NMDA	*N*-methyl-d-aspartate receptor
mGluRs	Metabotropic glutamate receptors
GABA	Gamma-aminobutyric acid
COMT	Catechol-*O*-methyl transferase
MGL	Magnesium low blood levels
MGH	Magnesium high blood levels
